# Spondylodiscitis and Spinal Epidural Abscess After Transoral Robotic Surgery Radical Tonsillectomy

**DOI:** 10.1002/oto2.61

**Published:** 2023-06-21

**Authors:** Jacquelyn K. Callander, Aaron J. Clark, William Dillon, William R. Ryan

**Affiliations:** ^1^ Department of Otolaryngology–Head and Neck Surgery University of California San Francisco San Francisco California United States; ^2^ Department of Neurological Surgery University of California San Francisco San Francisco California United States; ^3^ UCSF Department of Radiology Neuroradiology Section, University of California San Francisco San Francisco California United States

**Keywords:** oropharyngeal squamous cell carcinoma, spinal epidural abscess, spondylodiscitis, TORS, transoral robotic surgery

Over the past 15 years, transoral robotic surgery (TORS) has emerged as a powerful, less invasive tool to treat oropharyngeal cancers. The goal of surgical treatment is de‐escalation therapy to decrease treatment morbidity and improve quality of life outcomes while maintaining overall and disease‐free survival.^1^ This may involve treatment with definitive surgery and adjuvant radiation with avoidance of adjuvant chemotherapy, or even decreasing radiation dose.^2^ While typical complications after TORS are well‐reported in the literature, we describe a rare case of spondylodiscitis and spinal abscess treated with computed tomography (CT)‐guided needle aspiration and antibiotics. This case report met criteria for University of California San Francisco Institutional Review Board exemption.

## Case Report

**Figure 1:**
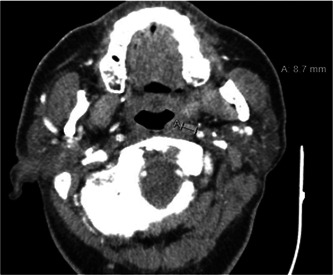
Preoperative computed tomography neck demonstrating left retropharyngeal lymph node (denoted by the letter “A”), positive for squamous cell carcinoma on surgical pathology.

A 65‐year‐old male with history of hypertension, gout, arthritis, and reflux presented with a cT1N1M0 left tonsil human papillomavirus positive squamous cell carcinoma with fine needle aspiration biopsy‐proven metastasis to the ipsilateral neck. CT chest and positron emission tomography (PET)‐CT did not identify distant metastatic disease. The patient underwent a TORS‐assisted left radical tonsillectomy, left selective neck dissection levels 1b, 2a, 2b, 3, and 4, and ligation of left facial and lingual arteries, without intraoperative complication. Uniquely, we also excised a single retropharyngeal lymph node, seen on preoperative CT neck, that intraoperatively was located just deep and posterior to the left superior constrictor muscle in a paramedian location (Figure 1). Postoperatively, the patient had severe dysphagia requiring nutritional support via nasogastric feeding tube for 3 weeks. Surgical pathology demonstrated negative margins, a positive retropharyngeal lymph node, and 3/32 positive neck lymph nodes without extranodal extension, perineural invasion, or lymphovascular invasion.

**Figure 2:**
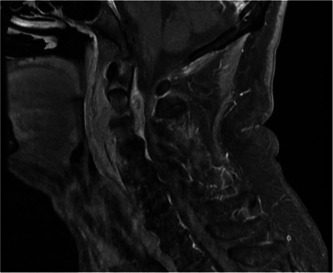
Magnetic resonance imaging cervical spine with contrast demonstrating C2‐3 discitis/osteomyelitis with epidural phlegmon and abscess causing spinal canal narrowing. A small focus of gas in the prevertebral phlegmon raises concern for fistulous communication with the oropharynx.

Four weeks postoperatively, a radiation‐planning CT neck demonstrated a C2‐3 epidural abscess. Magnetic resonance imaging (MRI) showed prevertebral phlegmon, C2‐3 spondylodiscitis, and epidural abscess spanning C1‐C3 (Figure 2). He was asymptomatic with intact neurologic examination. A small fistulous tract between the oropharynx the prevertebral site of the infection was identified on MRI, suggesting the mechanism of infection was direct extension from the oropharynx from the retropharyngeal lymph node excision. An anterior neck approach for drainage with cervical discectomy, debridement, and fusion was considered, as is customary for spinal epidural abscesses. Given the mucosal surgical defect would likely expand to a more extensive pharyngospinal fistula, this approach was thought to be unfavorable. Instead, a diagnostic and therapeutic posterior percutaneous CT‐guided aspiration of the abscess was performed. Based on cultures showing polymicrobial oral flora (*Streptococcus anginosus*, anaerobic gram‐negative rods), the patient underwent a 6‐week course of intravenous (IV) Ceftriaxone and IV Flagyl.

Repeat MRI 16 weeks postoperatively revealed marked interval improvement in the cervical discitis and prevertebral phlegmonous change. Subsequently, the patient received adjuvant radiation (60 Gray in 33 fractions) 3 months after drainage of the epidural abscess. On his 3‐month posttreatment CT and PET scans (6‐month posttreatment of epidural abscess), he was without evidence of disease or recurrent infection.

## Discussion

Few cases of spondylodiscitis with and without abscess after TORS radical tonsillectomy have been reported with high rates of mortality up to 43%.^3^, ^4^ Despite its rarity, it is of utmost relevance given the recent ORATOR 2 trial was stopped due of death from spondylodiscitis.^5^ A post‐TORS patient presenting with fevers, neck pain (including point tenderness of cervical vertebrae), upper extremity weakness, or paresthesia should be evaluated for spinal or paraspinal infection. Risk factors identified in the literature include neoadjuvant or adjuvant radiation therapy, long operative time, severe blood loss, and extensive soft‐tissue pharyngeal dissection.^4^ The latter may have been at play in the case presented due to the excision of the suspicious retropharyngeal lymph node, though the parapharyngeal space was not otherwise explored. Studies have suggested blood cultures or CT‐guided diagnostic fine needle aspiration biopsy to guide antibiosis with surgical drainage or debridement of an epidural abscess only in the setting of clinical signs of neural compression.^4^ Given the high rate of neurologic deterioration and mortality with this complication, we propose consideration of therapeutic CT‐guided drainage of epidural abscess even in the absence of clinical signs of compression, followed by culture‐directed IV antibiosis. The use of CT guidance permits for safe navigation of the complex anatomy in this region. This allowed our patient to avoid revision open surgery with potential free flap reconstruction. Multi‐institutional studies are necessary to further elucidate the prevalence and most appropriate treatment algorithm for this subset of infectious spinal lesions.

## Author Contributions

**Jacquelyn K. Callander**, conception and drafting of the manuscript, data acquisition; **Aaron J. Clark**, conception and critical review of the manuscript, direct involvement in care of the patient; **William Dillon**, conception and critical review of the manuscript, direct involvement in care of the patient; **William R. Ryan**, conception and critical review of the manuscript, direct involvement in care of the patient; All authors provided final approval of this manuscript.

## Disclosures

### Competing interests

William R. Ryan, MD, is on the Scientific Advisory Boards for Olympus and Rakuten Medical.

### Funding source

The authors have no other funding or financial relationships to disclose.
